# Optimization of AAV expression cassettes to improve packaging capacity and transgene expression in neurons

**DOI:** 10.1186/1756-6606-7-17

**Published:** 2014-03-11

**Authors:** Jun-Hyeok Choi, Nam-Kyung Yu, Gi-Chul Baek, Joseph Bakes, Daekwan Seo, Hye Jin Nam, Sung Hee Baek, Chae-Seok Lim, Yong-Seok Lee, Bong-Kiun Kaang

**Affiliations:** 1 Department of Biological Sciences, College of Natural Sciences, Seoul National University, Seoul 151-747, Korea; 2 Center for RNA Research, Institute for Basic Science, Seoul, Korea; 3 Department of Life Science, College of Natural Science, Chung-Ang University, Seoul 156-756, Korea; 4 Department of Biological Sciences, Creative Research Initiatives Center for Chromatin Dynamics, Seoul National University, Seoul 151-742, South Korea

**Keywords:** Adeno-associated virus, WPRE, SV40 late polyadenylation signal sequence, Neuron

## Abstract

Adeno-associated virus (AAV) vectors can deliver transgenes to diverse cell types and are therefore useful for basic research and gene therapy. Although AAV has many advantages over other viral vectors, its relatively small packaging capacity limits its use for delivering large genes. The available transgene size is further limited by the existence of additional elements in the expression cassette without which the gene expression level becomes much lower. By using alternative combinations of shorter elements, we generated a series of AAV expression cassettes and systematically evaluated their expression efficiency in neurons to maximize the transgene size available within the AAV packaging capacity while not compromising the transgene expression. We found that the newly developed smaller expression cassette shows comparable expression efficiency with an efficient vector generally used for strong gene expression. This new expression cassette will allow us to package larger transgenes without compromising expression efficiency.

## Background

Viral vectors are efficient tools for expressing transgenes in diverse cell types *in vitro* and *in vivo*. The type of virus should be selected with care, because they possess advantages and disadvantages, depending on the purpose of the research [1]. Adeno-associated virus (AAV) is known to be one of the most useful viral vectors for transgene delivery because it is not pathogenic, induces a minimal immune response, infects both dividing and non-dividing cells, and the transgene expression persists for a longer time in cells. The genomes of AAV vectors infected into the host cell exist primarily in an extrachromosomal state, not integrating into the host genome. They can be inserted into only a single specific integration site in the human genome, which may be an advantage for stable long-term expression without insertional mutagenesis. In addition, AAV can be readily produced in high titers and deliver transgenes with high efficiency. Adenovirus, herpes simplex virus (HSV), retrovirus, and lentivirus also have their advantages in transgene delivery. However, adenovirus and HSV are not suitable for the purpose of stable expression in non-dividing cells such as neurons, because they only allow transient expression. They also have the disadvantage of inducing high immune responses. Retroviruses only transduce dividing cells, and, in common with lentivirus, have a risk of insertional mutagenesis [1]. For these reasons, AAV vectors are frequently used in basic researches and are attractive candidates for use in gene therapy. AAV has also been the vector of choice in recent clinical trials of neurological diseases, including Parkinson's and Alzheimer's disease [2].

However, the use of AAV vectors is limited by their relatively small maximal packaging capacity [3,4]. Although there is a report describing specific serotypes with much larger packaging capacity [5], other studies [6-8] indicated that intact genomes were not, in fact, packaged in that case [3], and further suggested a strict size cutoff of 5.2 kb. Efforts have been made to expand the available AAV transgene size by trans-splicing and/or recombination to split the AAV expression cassette to two viral vectors [9]. However, the expression efficiency is still much lower than that of a single viral vector.

Despite these efforts, an AAV expression cassette capable of delivering a large transgene with high expression level is still not available. As a strategy to expand the available size of transgenes in AAV, we decided to minimize the size of the expression cassette to allow more space for transgenes. Using an AAV expression cassette known to express transgenes with high efficiency as a template, we generated a variety of shorter cassettes. Then we systematically evaluated their expression efficiency in neurons to maximize packaging capacity while not compromising transgene expression.

## Results

### Experimental strategy for modifying expression cassettes

**Figure 1:**
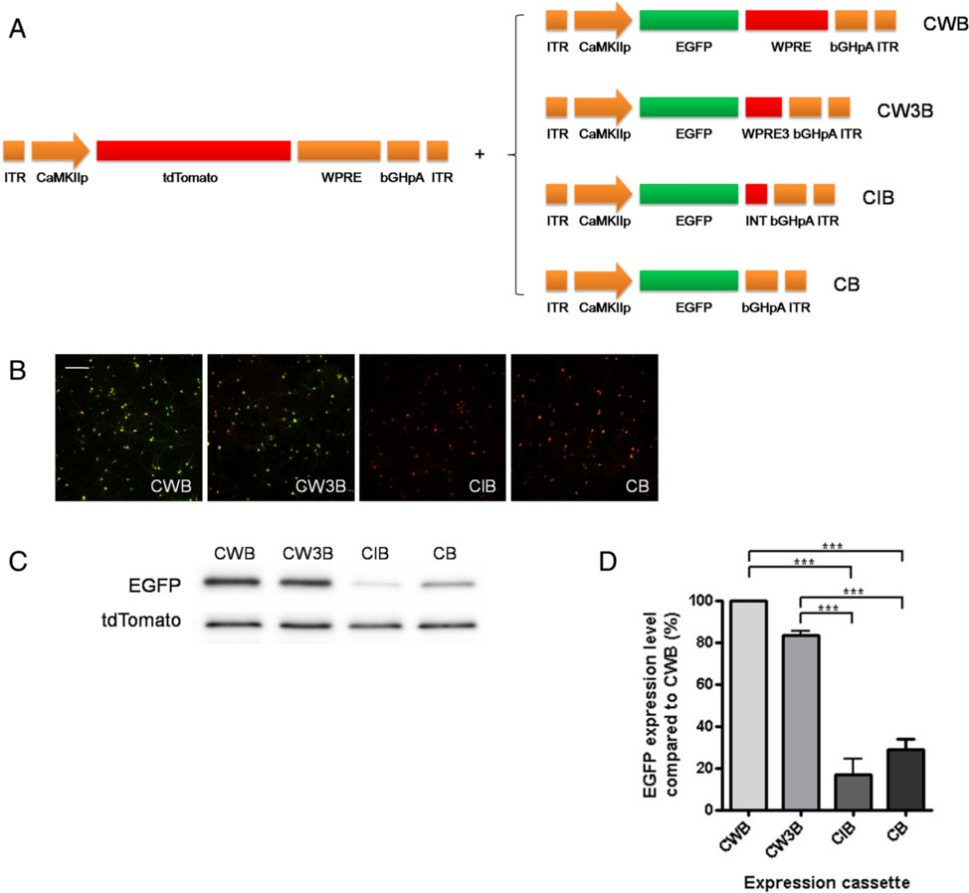
**Comparison of expression cassettes with modification or deletion of WPRE. A**. Schematic drawings of expression cassettes tested. Each EGFP expression cassette was transduced into hippocampal neuron cultures together with tdTomato expression cassette, which contains complete WPRE and bGHpA. (ITR, Inverted Terminal Repeat; CaMKIIp, CaMKIIα promoter; WPRE, Woodchuck hepatitis virus posttranscriptional regulatory element, bGHpA: bovine growth hormone polyadenylation signal, INT: chimeric intron) **B**-**C**. Representative images of cell fluorescence **(B)**, and western blot analysis of EGFP and tdTomato expression **(C)**. Scale bar, 200 μm. **D**. Summary graph of western blot analysis shows that the expression level of EGFP driven by CW3B having WPRE3 (247 bp) is comparable (83.4%) to CWB having WPRE (600 bp). In contrast, EGFP expression level was significantly reduced in CIB (16.6%, P < 0.001) and CB (28.6%, P < 0.001) compared to CWB. (***P < 0.001, ANOVA Bonferroni’s post-hoc comparison).

**Figure 2:**
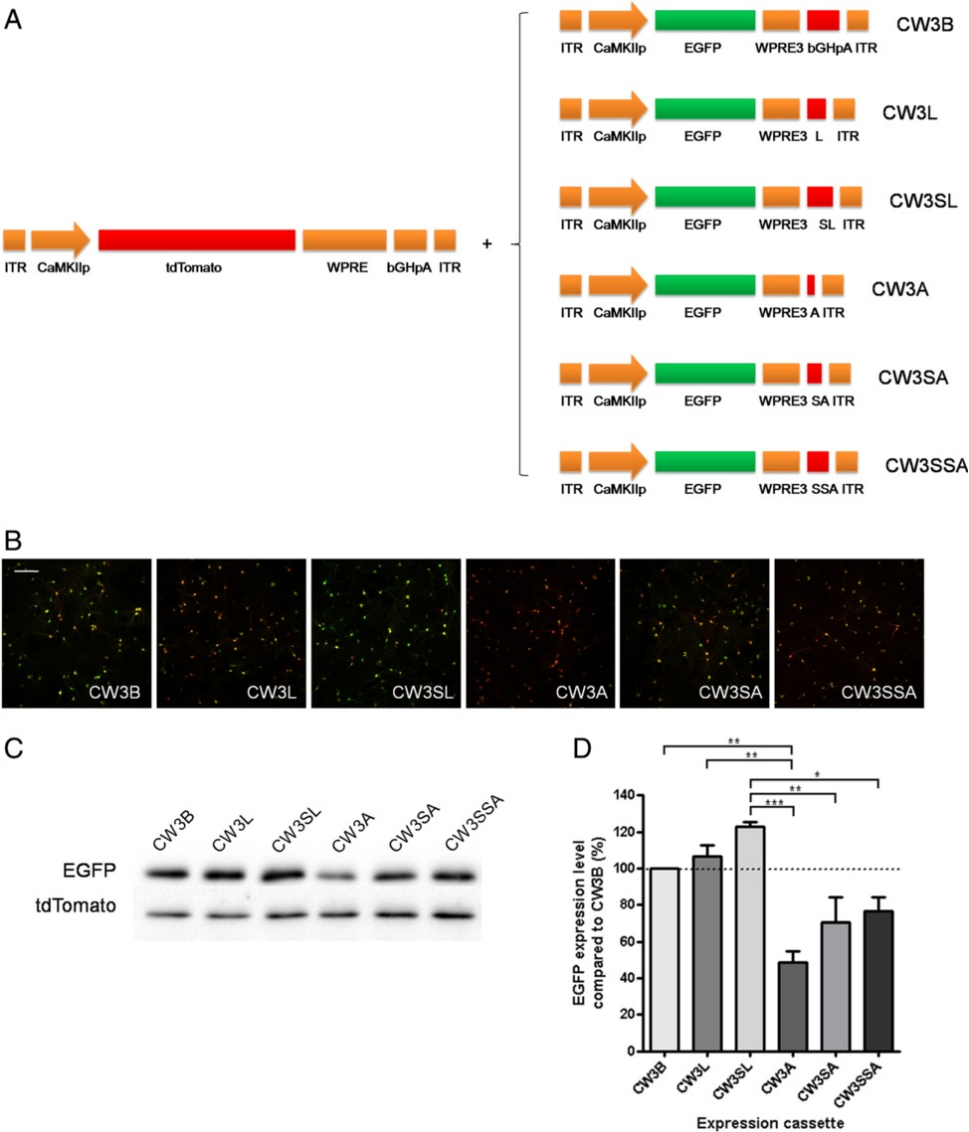
**Comparison of transgene expression with modification of polyadenylation signal. A**. Schematic drawings of EGFP expression cassettes having modifications in polyadenylation signal. (L: SV40 late polyadenylation signal; SL: SV40 late polyadenylation signal upstream element + SV40 late polyadenylation signal; **A**: synthetic polyadenylation signal, SA: SV40 late polyadenylation signal upstream element + synthetic polyadenylation signal; SSA: double copy of SV40 late polyadenylation signal upstream element + synthetic polyadenylation signal). **B**-**C**. Representative images of fluorescence **(B)** and western blot analysis **(C)** of EGFP and tdTomato expression. Scale bar, 200 μm. **D**. Summary graph shows that CW3SL containing WPRE3, an additional SV40 late polyadenylation signal upstream element followed by SV40 late polyadenylation signal, drives highest EGFP expression level. CW3B and CW3L drive significantly higher expression than CW3A (P < 0.01), while CW3SL drives significantly higher expression than CW3A (P < 0.001), CW3SA (P < 0.01), and CW3SSA (P < 0.05) (*P < 0.05, **P < 0.01, ***P < 0.001, ANOVA Bonferroni’s post-hoc comparison).

To generate a series of AAV expression cassettes, we used a strong expression cassette which contains neuron-specific promoter [10], transgene encoding enhanced green fluorescent protein (EGFP), woodchuck hepatitis posttranscriptional regulatory element (WPRE) [11-13], and bovine growth hormone polyadenylation signal (bGHpA) [14,15] as an initial template. In this expression cassette called CWB (*C*aMKII, *W*PRE, *b*GHpA), three elements were fused with minimal sized DNA linker sequences (6 bp for each junction). First, we modified the CWB cassette by replacing WPRE with shorter elements and examined transgene expression efficacy of the modified cassettes (Figure 1). Next, we generated further modified cassettes by replacing bGHpA with shorter polyadenlyation signal sequences and examined their transgene expression efficiency (Figure 2). Each AAV vector expressing EGFP encoded by the modified expression cassettes was transduced into cultured hippocampal neurons together with control AAV expressing a red fluorescent protein, tdTomato by the CWB cassette (CWB-tdTomato). Then, we performed western blot analysis by using antibodies against EGFP and tdTomato to quantify normalized EGFP expression.

### Transgene expression driven by AAV expression cassettes with modified WPRE

WPRE is a 600 bp long tripartite element containing gamma, alpha, and beta elements, in the given order [16] and contributes to the strong expression of transgenes in AAV systems [11-13]. It also enhances the expression of a transgene lacking introns. In a previous study [17], a shortened WPRE sequence (WPRE2) containing a minimal gamma element and a partial alpha-beta element was used in an AAV vector, but its transgene expression efficiency was not quantitatively examined. We shortened this sequence further so that it contained only minimal gamma and alpha elements (WPRE3). Introns are known to possess a posttranscriptional regulatory element that efficiently induces transport of mRNA out of the nucleus and enhances mRNA stability [18]. Therefore, we inserted a chimeric intron sequence instead of WPRE to examine whether an intron sequence, shorter than WPRE, could increase transgene expression comparable to WPRE. We compared the EGFP expression efficiency of expression cassettes containing WPRE (CWB), WPRE3 (CW3B), or a short chimeric intron (CIB), together with an expression cassette lacking any of these (CB) (Figure 1). CB that lacks WPRE from CWB exhibited reduced EGFP expression compared to CWB (28.6% of CWB), indicating that WPRE is critical for strong expression. The chimeric intron did not enhance the expression of EGFP. The EGFP expression level of CW3B was not statistically different compared with that of the control CWB cassette (83.4%), even though the size of WPRE had been significantly reduced (from 600 bp WPRE to 247 bp WPRE3, approximately 41.2% of original size).

### Effects of modification in the polyadenylation signal on transgene expression

Using CW3B as a template, we next compared the transgene expression efficiency of various polyadenylation signal sequences (Figure 2A). Aside from the CWB component bGHpA, the SV40 early/late polyadenylation signal is also commonly used for efficient transgene expression in mammalian cells. Because the late polyadenylation signal sequence acts more efficiently for transgene expression than the early polyadenylation signal sequence due to the presence of a short upstream element preceding the mRNA cleavage site [19,20], only the late polyadenylation signal sequence was tested in this study. We first designed an expression cassette containing the SV40 late polyadenylation signal sequence instead of bGHpA (CW3L). A previous study using lentiviral vectors showed that the upstream element in the late SV40 polyadenylation signal enhances the function of the polyadenylation signal to a greater extent in a tandem arrangement than when alone [21]. Therefore, we tested this in our study as well, by adding an additional upstream sequence element prior to the full late polyadenylation signal (CW3SL). Because a short synthetic polyadenylation signal was successfully used in a previous study [22], we also generated AAV expression cassettes that have a synthetic polyadenylation signal alone (CW3A), or together with a single (CW3SA) or tandem upstream element of the SV40 late polyadenylation signal (CW3SSA). Compared with CW3B, the expression levels of EGFP were 106.5% by CW3L, 122.7% by CW3SL, 49.7% by CW3A, 70.3% by CW3SA, and 76.4% by CW3SSA (Figure 2B-D).

### Effect of cassette size on transgene expression efficiency

**Figure 3:**
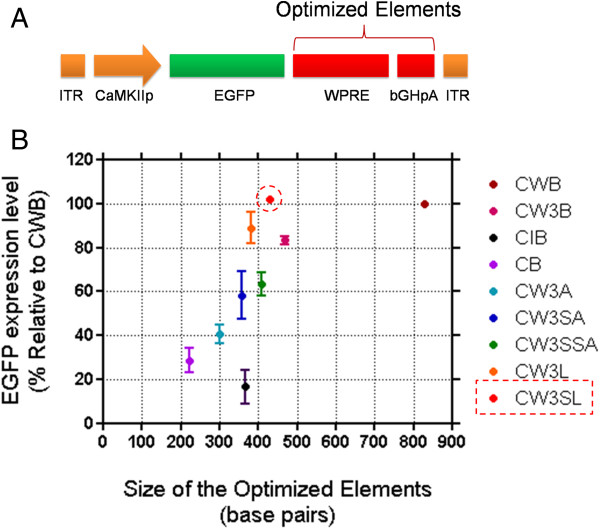
**The plot of optimized elements’ size against transgene expression efficiency of each expression cassette. A**. Common scheme of the expression cassette generation in this study. The target element sites varied for optimization are indicated. **B**. The normalized EGFP expression level by each cassette plotted against the size of each expression cassette with modification in WPRE and bGHpA sites. Note that size reduction of about 399 bp in these two elements in CW3SL still gives similar level of transgene expression as that of CWB.

We plotted the normalized EGFP expression level against the sequence size of the posttranscriptional regulatory elements and polyadenylation signals (excluding ITRs, promoters, and transgenes, which are constant for all expression cassettes; Figure 3). Although the EGFP expression level driven by the expression cassettes increased linearly as the size of elements increased, CW3SL and CW3L showed higher levels of EGFP expression compared to their size. In particular, CW3SL produced comparable EGFP expression to the parental CWB (103.4%), despite the fact that CW3SL is substantially smaller than CWB in size.

### Transgene expression by CW3SL in mice brain

**Figure 4:**
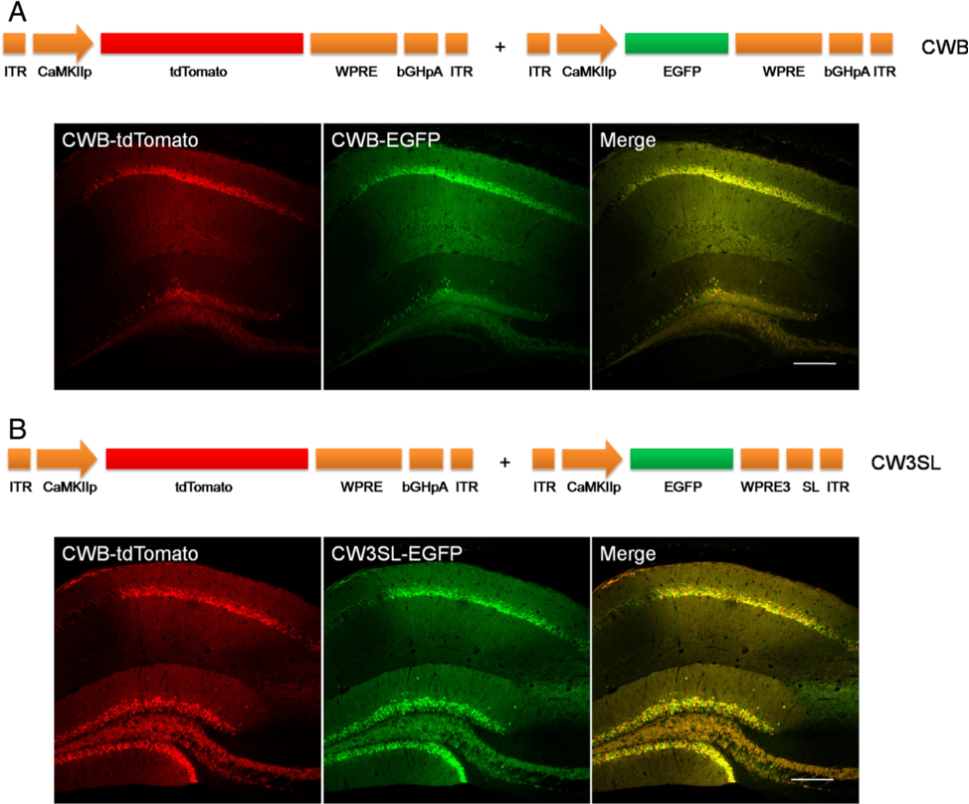
**Transgene expression in the mouse brains with CW3SL expression cassette.** Either CWB-EGFP **(A)** or CW3SL-EGFP **(B)** was injected together with CWB-tdTomato into the hippocampal CA1 region. The expression levels of EGFP and tdTomato were detected by confocal fluorescence imaging. Scale bar, 200 μm.

We next examined whether CW3SL drove transgene expression efficiently *in vivo*. Either CWB-EGFP (Figure 4A) or CW3SL-EGFP (Figure 4B) was injected with CWB-tdTomato into the hippocampal CA1 region in mice. The EGFP fluorescence intensity was normalized by the tdTomato fluorescence intensity in the expressed CA1 area to measure the expression efficiency of each expression cassette. CW3SL-EGFP showed a similar level of expression to CWB-EGFP, although it was slightly weaker (86.2%).

### CW3SL significantly increases AAV packaging limit of transgene

**Figure 5:**
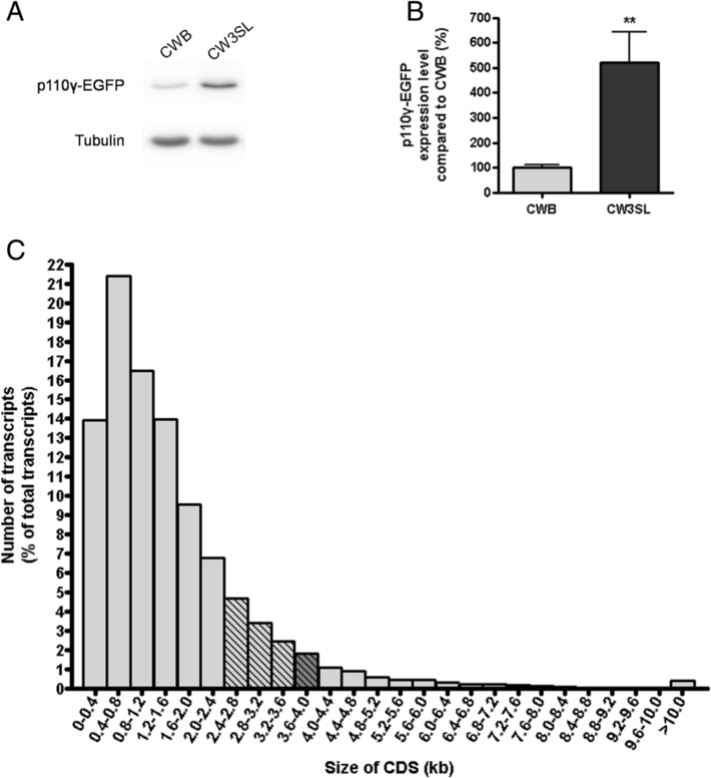
**Utilization of CW3SL expression cassette for large transgene expression. A**. Western blot analysis of p110γ-EGFP fusion protein expression by CWB-p110γ-EGFP and CW3SL-p110γ-EGFP. **B**. Quantification of the western blot analysis. p110γ-EGFP expression level was normalized to that of tubulin, which was then normalized to the average expression level by CWB-p110γ-EGFP. **C**. The percentage of transcripts in mouse hippocampus with coding sequence length within every 0.4 kb. Transcripts expressed in mouse hippocampus were selected based on RNA sequencing data (analyzed based on Ensembl Genes version 67, NCBIM37). The transcripts in the range of 2.4-4 kb (striped bars) and those in the range of 3.6-4 kb (darker and striped bar) that exceed the packaging capacity of conventional AAV expression cassettes and CWB cassette, respectively, become available using CW3SL cassette.

We next examined the capability of the CW3SL expression cassette for packaging larger transgenes that exceed the packaging limit of CWB. We fused the DNA encoding p110γ, a phosphatidylinositol-4,5-bisphosphate 3-kinase catalytic subunit gamma isoform, with EGFP, which resulted in 4.03 kb long DNA. When we cloned this fused DNA into the CWB cassette, the size of DNA to be packaged is 5.6 kb, which exceeds the 5.2 kb packaging limit for AAV. However, the DNA length of CW3SL-p110γ-EGFP to be packaged in AAV is 5.2 kb, which allows successful AAV production. Although CWB-p110γ-EGFP did produce comparable amount of virus (6.61 × 10^12^ GC/ml for CWB-p110γ-EGFP, 7.01 × 10^12^ GC/ml for CW3SL-p110γ-EGFP; GC: genome copy), CW3SL drove expression of p110γ-EGFP five times more compared to CWB, demonstrating that this novel expression cassette CW3SL improves AAV packaging capacity for larger transgenes (Figure 5A-B).

## Discussion

AAV offers many advantages as a gene delivery system over other viral vectors; however, its strict packaging capacity limits its use for delivering large transgenes or large cell-type specific promoters. Techniques to divide a gene into two AAV vectors and then combining each segment to reconstitute the full-length sequence after transduction by trans-splicing and recombination have been developed, but this technique is still in its infancy showing much lower expression efficiency compared to a non-divided single vector [9]. Other groups have also developed shorter expression cassettes containing a functional, minimal-sized polyadenylation signal [15,22]; however, we found that they yield only very low levels of transgene expression. To fully exploit the potential of AAV vectors, a short expression cassette capable of supporting proper expression levels of transgenes required for basic and clinical applications is highly required.

In this study, we systematically compared AAV expression cassettes comprising various short regulatory elements as substitutes for the WPRE and polyadenylation signal of a prototype cassette capable of expressing high levels of transgenes. We found that a shortened WPRE, named WPRE3, which contains two of the three regulatory elements of WPRE, affords efficient transgene expression (83.4%) even when reduced in size from 600 bp to 247 bp.

We then altered the polyadenylation signal of the modified expression cassette (CW3B) that contains WPRE3. Because synthetic, minimal consensus polyadenylation signal sequence was used successfully in conjunction with a strong promoter in a previous study [22], we used this 49 bp segment in AAV in conjunction with the CaMKIIα promoter (CW3A); however, the resulting transgene expression driven by CW3A was only 40.4% of the original expression cassette CWB. The SV40 early/late polyadenylation signal is often used for expressing the transgene in mammalian cells. It is well known that SV40 late polyadenylation signal is more efficient due to a short upstream element sequence [19,20]. This sequence is also known to increase the efficiency of a weak polyadenylation signal and even more so when used in tandem [21]. In the present study, we found that the 135 bp SV40 late polyadenylation signal (CW3L) showed similar efficiency to that of bGHpA, which is 223 bp (CW3B). An additional upstream element sequence (CW3SL) further improved the efficiency and restored the reduced level of expression from WPRE3. The upstream element also improved the efficiency of the synthetic polyadenylation signal (comparing CW3A, CW3SA, and CW3SSA). However, these expression cassettes were not as efficient in terms of size as the ones containing the SV40 late polyadenylation signal (CW3L and CW3SL).

The new CW3SL cassette provided 399 bp additional cloning capacity compared with the original template vector CWB and produced comparable expression to CWB (103.4%). The maximal packaging capacity of CWB and CW3SL is approximately 3.6 kb and 4 kb respectively. Among the transcripts expressed in the mouse hippocampus (defined by RNA sequencing), 1.82% (488 genes) have coding sequence length within the range of 3.6 to 4 kb (Figure 5C). Furthermore, note that CWB represents an optimized, compact expression cassette from which sequences not required for expression have been eliminated in our study. Conventionally used AAV backbones which include a commonly used neuronal expression cassette composed of 1.3 kb CaMKIIα promoter (which shows comparable expression efficiency with 0.4 kb CaMKIIα promoter [10] used in CWB), WPRE, and human growth hormone polyadenylation signal [23-25] has a capacity of 2.4 kb. Therefore, when compared with these conventionally used AAV cassettes, the CW3SL construct would obtain even more cloning capacity than 399 bp. 12.40% (3324 genes) of the transcripts expressed in the mouse hippocampus have coding sequence length within the range of 2.4 to 4 kb. Therefore, CW3SL now makes it possible to express larger transgenes at high efficiency compared with the prototype strong expression cassette CWB and other conventional expression cassettes. In some cases, CW3SL may provide an opportunity to package larger transgenes in AAV vectors that were previously impossible to package, while in other cases it may enable fusing a reporter such as EGFP or other fluorescent proteins without losing expression efficiency as we demonstrated. The family of new expression cassettes tested in this study could also provide a variety of cloning options. Other cassettes shorter than CW3SL would allow packaging of even longer transgenes and still would be useful in certain experimental conditions although their expression level would be lower than that of CW3SL. The new expression cassettes described here would also be useful for designing other derivatives. For example, the SV40 late polyadenylation signal supported the expression of transgenes as efficiently as vectors containing bGHpA in the presence of WPRE3 (Figure 2). Thus, the SV40 late polyadenylation signal alone may support expression at a level similar to that of the CB expression cassette while saving 88 bp. In addition, an SV40 late polyadenylation signal upstream element may further enhance the efficiency of transgene expression. In this study, we used the CaMKIIα promoter to drive transgene expression in neurons, but it would also be possible to substitute this promoter with other ubiquitous promoters, cell-type–specific promoters, or even conditional promoters for different applications.

## Conclusions

One of the most critical limitations for application of AAV is the strict packaging capacity. To overcome this issue we systematically developed and compared various expression cassettes. We found that the newly developed smaller expression cassette shows comparable transgene expression efficiency with the efficient vector generally used for strong transgene expression in AAV system. This new expression cassette will allow us to package larger transgenes without compromising expression efficiency.

## Methods

### Plasmid construction

The expression cassettes tested in this study are shown in Figures 1 and 2. Each element was PCR amplified and inserted into the AAV vector pAAV-Ef1a-DIO-hChR2(H134R)-EYFP-WPRE-pA, replacing the DNA sequences between the inverted terminal repeats (ITRs). The backbone plasmid and a CaMKIIα promoter (0.4 kb) were gifts from K. Deisseroth and T. Kaneko [26], respectively. WPRE was amplified from pLentilox3.1, and WPRE3 was produced by using recombinant PCR, in which the minimal sequences of γ and α elements were fused [16]. bGHpA was amplified from pcDNA3.1. A chimeric intron (5´-donor site from a human β-globin intron and 3´-acceptor site from an immunoglobulin heavy chain variable region intron) and the SV40 late polyadenylation signal were amplified from pZac2.1. The complete plasmid sequences are deposited in GenBank (GenBank accession number KJ411911-KJ411919).

### AAV production

AAVs (serotype 1) were purified from HEK293T cells that were transfected with plasmids containing each expression cassette, p5E18-RXC1, pAd-ΔF6 [27] (20 ml medium [DMEM/10% FBS] in a 150-mm culture dish). Three days after transfection, the medium containing AAV particles was collected and treated with Benzonase® (50 U/ml for 30 min at 37°C). After 1,500 rpm, 10 min centrifugation, the supernatant was loaded onto iodixanol gradients (54% 4 ml, 40% 5 ml, 25% 5 ml, and 15% 6 ml). Four milliliters of the 40% layer was collected after ultracentrifugation (69000 rpm, 70Ti rotor). The solution was exchanged with PBS and concentrated using an Amicon Ultra-15 centrifugal filter unit. The titer was measured using quantitative RT-PCR.

### Viral infection and western blot analysis

AAVs expressing EGFP and tdTomato were co-infected to DIV9 cultured hippocampal neurons derived from E18 rat embryos (5 × 10^8^ GC/well for a 12-well plate) and incubated at 37°C in a CO_2_ incubator for 9 days. Cells were then collected after brief washing with 1× PBS and lysed with RIPA buffer (50 mM Tris-Cl, pH 7.6, 150 mM NaCl, 1 mM EDTA, 1% NP-40, 0.25% SDS, 0.5% sodium deoxycholate) containing PIC (Protease Inhibitor Cocktail, Roche). The cell lysate was incubated on ice for 10 min, and SDS-gel loading buffer (50 mM Tris-Cl, pH 6.8, 100 mM dithiothreitol, 2% SDS, 0.1% bromophenol blue, 10% glycerol) was added to each sample. For electrophoresis, equal amount of proteins were loaded onto 12% polyacrylamide gels. Separated proteins were then transferred onto a nitrocellulose membrane at 4°C for 15–20 h. After blocking for 1 h with 5% skim milk in Tris-buffered saline containing 0.1% Tween-20 (TBST) at room temperature, the membranes were incubated with antibodies against either EGFP (1:5,000 NeuroMab), red fluorescent protein (RFP; 1:10,000, Rockland) or tubulin (1:100,000), at 4°C overnight. HRP-conjugated secondary antibodies were applied for 1 h at room temperature after washing with TBST. Enhanced chemiluminescence (Millipore) was used to detect antigen-antibody complexes. Images were acquired using a ChemiDoc XRS + System (Bio-Rad), and the densities of the bands were measured using the Image Lab program (Bio-Rad). For western blot analysis in Figures 1 and 2, the EGFP expression level of each expression cassette was normalized with tdTomato expression level. For western blot analysis in Figure 5, EGFP-p110γ levels were first normalized with tubulin levels, then normalized with the average of the level for CWB.

### Stereotactic viral injection and imaging

All surgical procedures were conducted under sterile conditions and approved by the Institutional Animal Care and Use Committee of Seoul National University. Wild-type C57BL/6 male mice (8 weeks of age) were anesthetized by i.p. injection of Zoletile and arranged in a stereotactic frame (Stoelting Co.). The hippocampal CA1 regions (AP: −1.8 mm, ML: ± 1.5 mm, DV: −1.7 mm) were targeted, and an equivalent amount of AAVs (EGFP: 8 × 10^8^ GC; tdTomato: 4 × 10^8^ GC) were delivered by using a 10-μL syringe pump (Hamilton Co.) at 6.0 μL/h. After an additional 10 min of diffusion, a needle was withdrawn and the animal’s scalp was sutured with black silk. After 3 weeks, the brain was removed and fixed in 4% paraformaldehyde at 4°C overnight, followed by dehydration in 30% sucrose at 4°C for 2 days. Sections (50 μm) were prepared by using a cryostat and mounted on glass slides with Vectashield (Vector Lab). Fluorescence images from hippocampal CA1 region were collected using a confocal microscope and analyzed with ImageJ program. To measure the EGFP expression efficiency from the virus-infected area, tdTomato-expressed CA1 pyramidal cell body layer was selected from each image, and the fluorescence intensities of EGFP and tdTomato of the region were measured. EGFP intensity was divided by tdTomato intensity to normalize the virus infusion efficiency.

## Competing interests

The authors declare that they have no competing interests.

## Authors’ contributions

JHC and NKY designed and performed experiments and wrote the first draft. GCB performed experiments. SHB and HJN provided hippocampal RNA sequencing data and DS analyzed these data. JB, CSL and YSL revised the manuscript critically for important intellectual contents. BKK conceptualized the hypothesis, supervised research and finalized the manuscript. All authors read and approved the final manuscript.
